# Supplementary material for: INRI-seq enables global cell-free analysis of translation initiation and off-target effects of antisense inhibitors
Source: Nucleic Acids Res. 2022 Oct 14;50(22):e128. doi: 10.1093/nar/gkac838 (PMC9825163; doi:10.1093/nar/gkac838)
Supplement: gkac838_Supplemental_Files [file gkac838_supplemental_files.zip › Supplementary Figures S1-6.pdf]

SUPPLEMENTARY FIGURES

INRI-seq enables global cell-free analysis of translation initiation and off-target effects of antisense inhibitors

Jens Hör, Jakob Jung, Svetlana Đurica-Mitić, Lars Barquist & Jörg Vogel

Figure S1

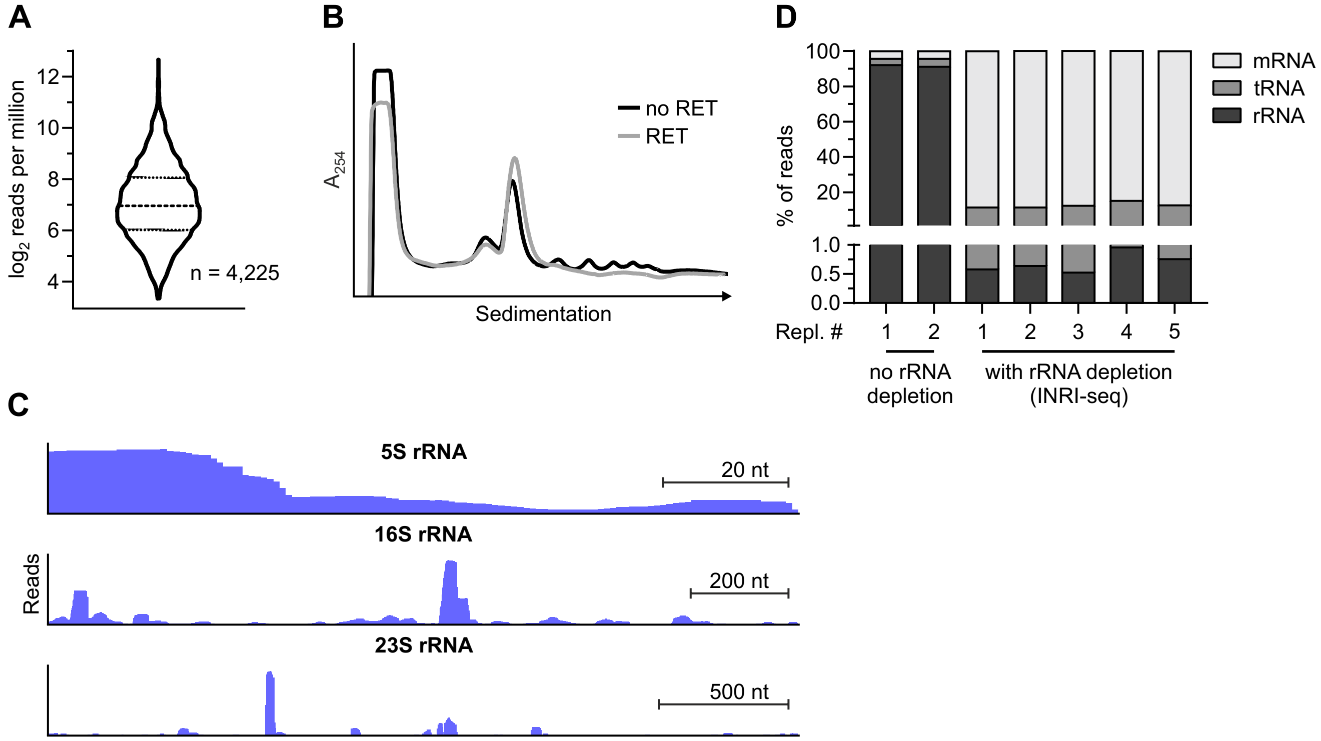
A
log2 reads per million
12
10
8
6
4
n = 4,225
B
A254
no RET
RET
Sedimentation
D
100
80
60
40
20
% of reads
1.0
0.5
0.0
mRNA
tRNA
rRNA
Repl. #
1
2
1
2
3
4
5
no rRNA depletion
with rRNA depletion (INRI-seq)
C
5S rRNA
20 nt
16S rRNA
Reads
200 nt
23S rRNA
500 nt

Figure S1. Quality control of INRI-seq.

(A) Violin plot showing the read distribution of all transcripts in the synthetic transcriptome with > 10 reads per million. The dashed line represents the median and the dotted lines represent the 25% and 75% percentiles.

(B) Sucrose gradient UV profiles of the sedimentation of *in vitro* translation reactions with or without addition of RET.

(C) Read distribution of rRNA genes without rRNA depletion.

(D) Comparison of the read distribution of RNA classes with and without rRNA depletion.

Figure S2

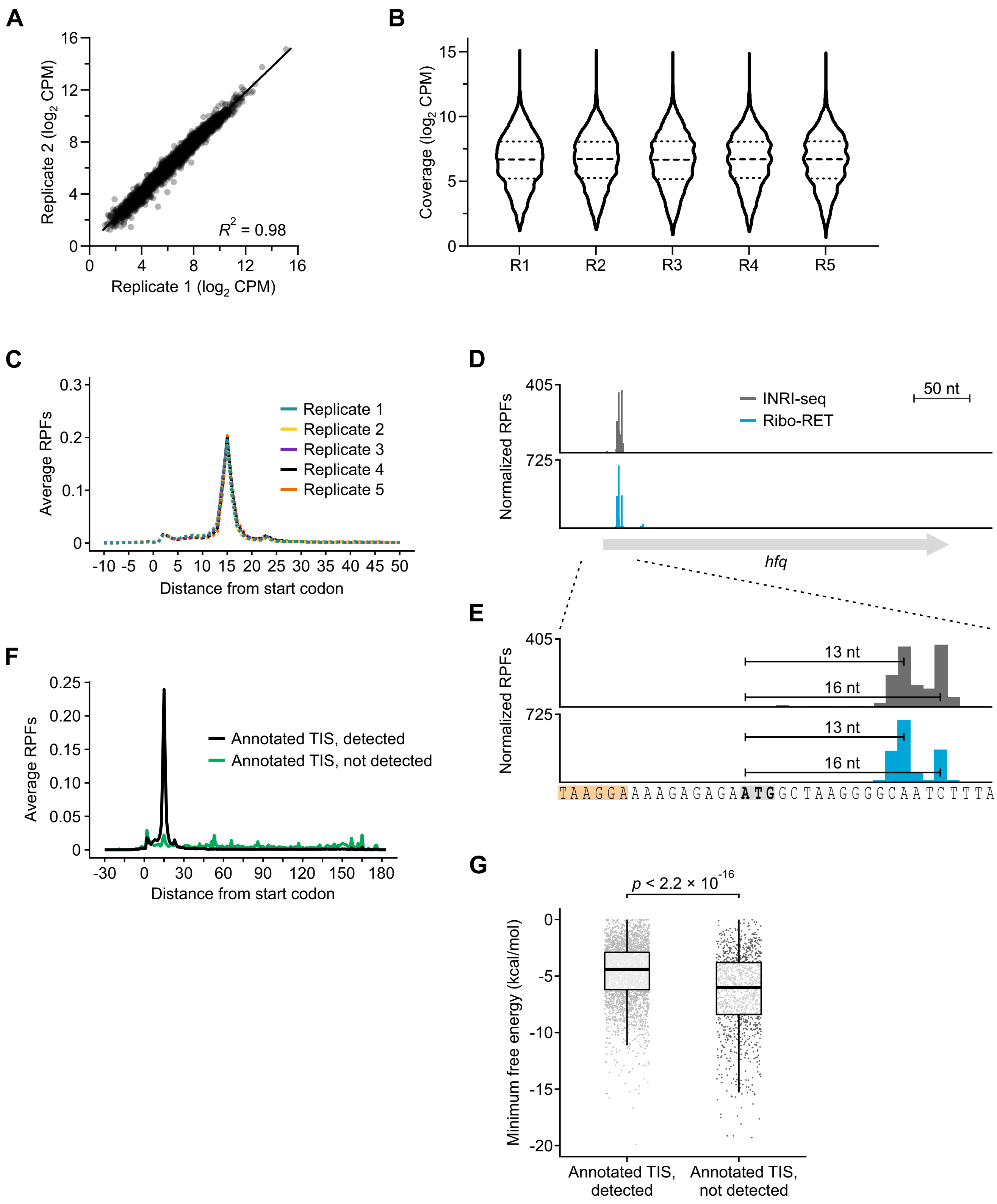
A
Replicate 2 (log2 CPM)
Replicate 1 (log2 CPM)
R² = 0.98
B
Coverage (log2 CPM)
R1
R2
R3
R4
R5
C
Average RPFs
Replicate 1
Replicate 2
Replicate 3
Replicate 4
Replicate 5
Distance from start codon
D
Normalized RPFs
405
725
INRI-seq
Ribo-RET
50 nt
hfq
E
13 nt
16 nt
TAAGGAAAAGAGAGAATGGCTAAGGGGCAATCTTTA
F
Annotated TIS, detected
Annotated TIS, not detected
G
p < 2.2 × 10⁻¹⁶
Minimum free energy (kcal/mol)
Annotated TIS, detected
Annotated TIS, not detected

Figure S2. Reproducibility of INRI-seq.

(A) Scatter plot showing the correlation between two representative replicates. R^2 represents the coefficient of determination.

(B) Violin plots showing the read coverage of the 4,149 transcripts detected in all five replicates (R1-R5). The dashed lines represent the medians and the dotted lines represent the 25% and 75% percentiles.

(C) The average distance of RPF peak density from annotated start codons is 15 nt for each of the replicates, and their RPF densities are congruent.

(D, E) RPF density at the TIS of *hfq*.

(F) The peak density of annotated TISs that INRI-Seq detected is 15 nt from the annotated start codon, while annotated TISs that were not detected have no clear density peak at 15 nt.

(G) Box-and-whisker plots of the minimum free energy (MFE) of predicted RNA folding around the TISs (-30 to +15 nt with respect to the start codon). The MFE of annotated TISs detected by INRI-seq is higher (median -4.4 kcal/mol) than for annotated TISs not detected by INRI-seq (median -6.0 kcal/mol). Boxes show 25th and 75th percentiles with the medians depicted as horizontal lines. Whiskers extend until 1.5 times the inter-quartile ranges. *p*-values were calculated using a Wilcoxon signed rank test.

Figure S3

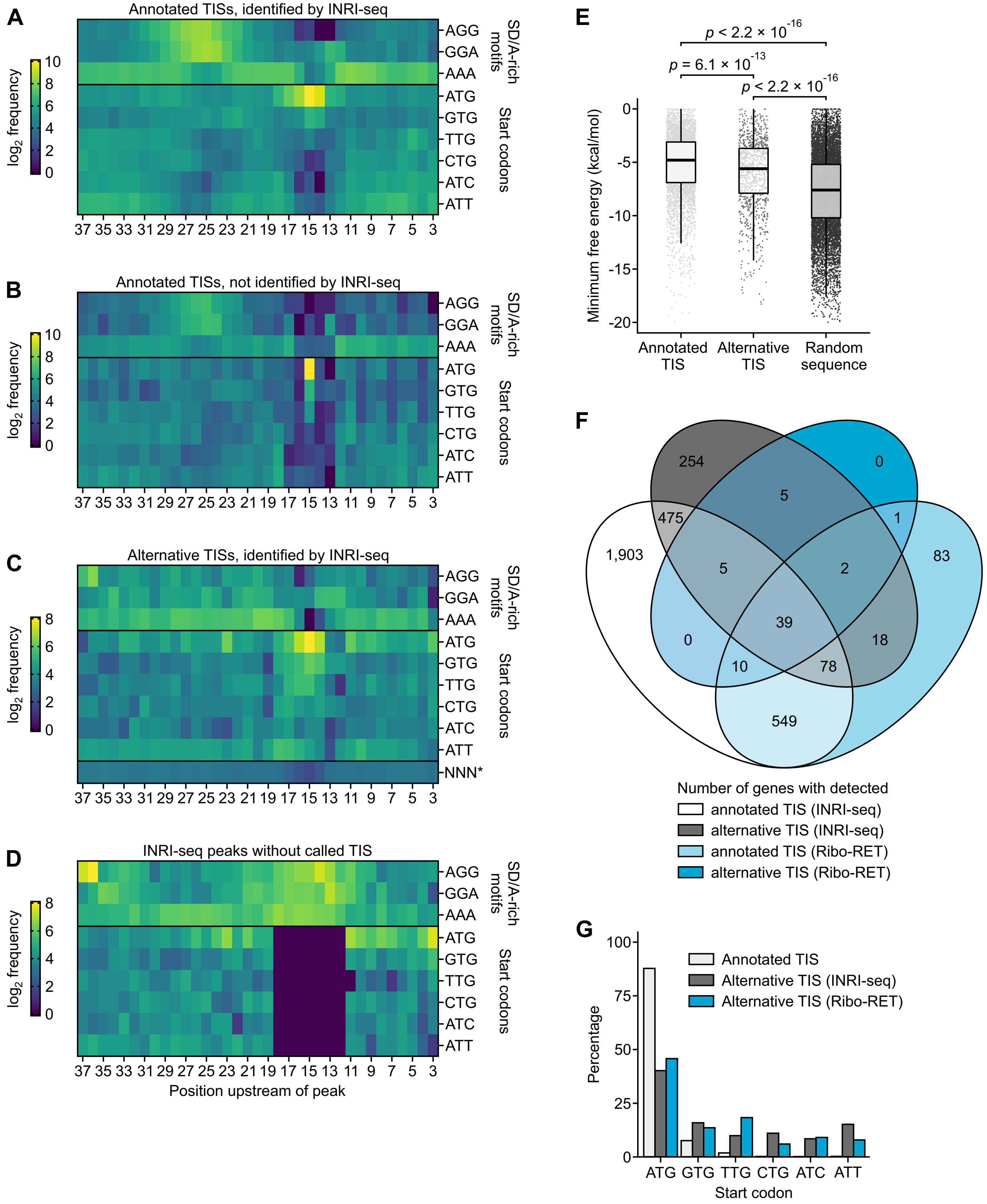

A
Annotated TISs, identified by INRI-seq
B
Annotated TISs, not identified by INRI-seq
C
Alternative TISs, identified by INRI-seq
D
INRI-seq peaks without called TIS
log2 frequency
AGG
GGA
AAA
ATG
GTG
TTG
CTG
ATC
ATT
NNN*
SD/A-rich motifs
Start codons
37 35 33 31 29 27 25 23 21 19 17 15 13 11 9 7 5 3
Position upstream of peak
E
p < 2.2 × 10-16
p = 6.1 × 10-13
p < 2.2 × 10-16
Minimum free energy (kcal/mol)
Annotated TIS
Alternative TIS
Random sequence
F
254
0
5
475
1
1,903
5
2
83
39
0
18
10
78
549
Number of genes with detected
annotated TIS (INRI-seq)
alternative TIS (INRI-seq)
annotated TIS (Ribo-RET)
alternative TIS (Ribo-RET)
G
Percentage
Annotated TIS
Alternative TIS (INRI-seq)
Alternative TIS (Ribo-RET)
ATG GTG TTG CTG ATC ATT
Start codon

Figure S3. Analysis of putative new TISs identified by INRI-seq.

(A) Heatmap showing the frequency of SD/A-rich motifs and start codons in proximity to the peaks of annotated TISs detected by INRI-seq.

(B) Heatmap showing the frequency of SD/A-rich motifs and start codons in proximity to the peaks of annotated TISs not detected by INRI-seq.

(C) Heatmap showing the frequency of SD/A-rich motifs and start codons in proximity to the peaks of alternative TISs as identified by INRI-seq. Additionally, the average distribution of non-start codon trinucleotides (NNN*) is shown. NNN* excludes the trinucleotides NAT and TGN, where N is any nucleotide, since these trinucleotides would show strong enrichment around the ATG start codon.

(D) Heatmap showing the frequency of SD/A-rich motifs and start codons in proximity to the INRI-seq peaks without called TIS.

(E) Box-and-whisker plots of the minimum free energy (MFE) of predicted RNA folding around the TISs (-30 to +15 nt with respect to the start codon). The MFE of annotated TISs is slightly higher (median -4.8 kcal/mol) than for alternative TISs identified by INRI-seq (median -5.6 kcal/mol). Randomly generated sequences of the same length show much stronger folding (median -7.7 kcal/mol) than both the annotated and the alternative TISs identified by INRI-seq. Boxes show 25th and 75th percentiles with the medians depicted as horizontal lines. Whiskers extend until 1.5 times the inter-quartile ranges. p-values were calculated using a Wilcoxon signed rank test.

(F) Venn diagram showing the number of genes for which the annotated or an alternative TIS was detected by INRI-seq or Ribo-RET. For some genes, both the annotated and an alternative TIS were detected, resulting in an overlap between these datasets.

(G) Barplot showing the percentages of identified start codon types for annotated TISs as well as alternative TISs identified by INRI-Seq and Ribo-RET.

Figure S4

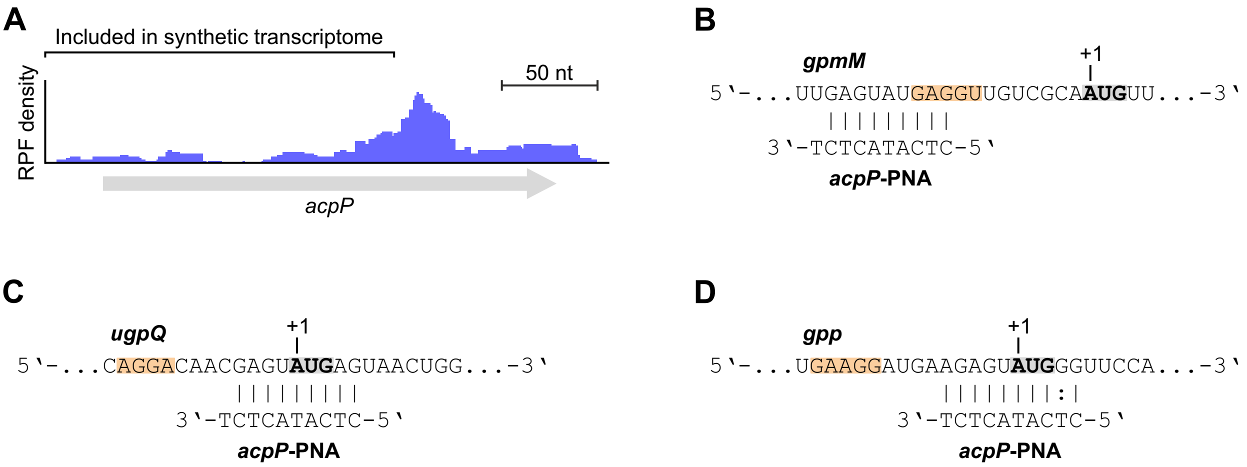
A
Included in synthetic transcriptome
50 nt
RPF density
acpP
B
gpmM
+1
5'-...UUGAGUAUGAGGUUGUCGCAAUGUU...-3'
|||||||||
3'-TCTCATACTC-5'
acpP-PNA
C
ugpQ
+1
5'-...CAGGACAACGAGUAUGAGUAACUGG...-3'
|||||||||
3'-TCTCATACTC-5'
acpP-PNA
D
gpp
+1
5'-...UGAAGGAUGAAGAGUAUGGGUUCCA...-3'
||||||||:|
3'-TCTCATACTC-5'
acpP-PNA

Figure S4. Evaluation of PNA off-targets.

(A) RPF distribution of *acpP* without addition of RET. The part of *acpP* included in the synthetic transcriptome is indicated.

(B-D) Regions of the *gpmM*, *ugpQ* and *gpp* transcripts targeted by *acpP*-PNA. Gray and bold, start codon. Orange, SD sequence.

Figure S5

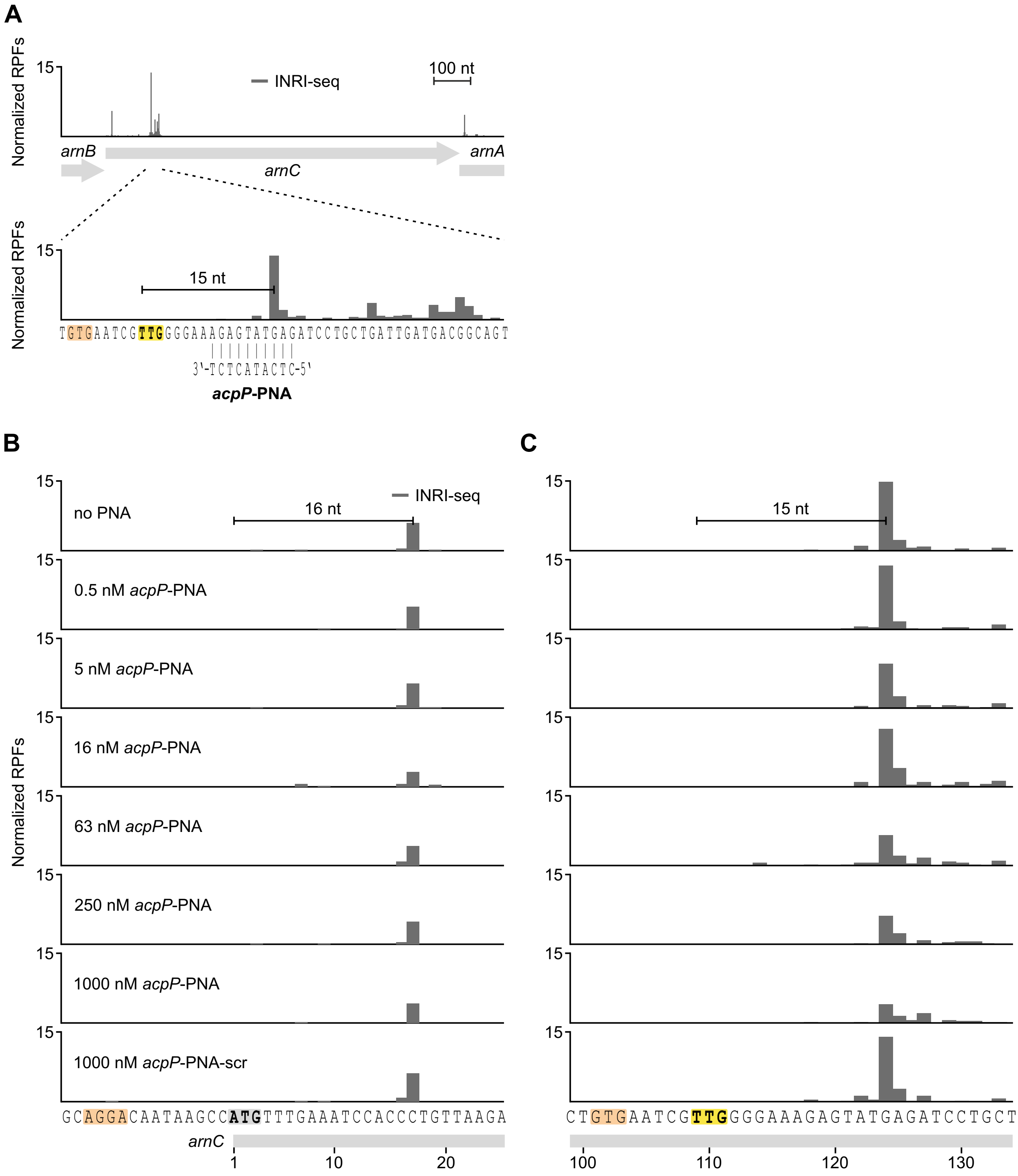

A
Normalized RPFs
15
INRI-seq
100 nt
arnB
arnC
arnA
15 nt
TGTGAATCGTTGGGGAAAGAGTATGAGATCCTGCTGATTGATGACGGCAGT
3'-TCTCATACTC-5'
acpP-PNA
B
C
16 nt
15 nt
no PNA
0.5 nM acpP-PNA
5 nM acpP-PNA
16 nM acpP-PNA
63 nM acpP-PNA
250 nM acpP-PNA
1000 nM acpP-PNA
1000 nM acpP-PNA-scr
GCAGGACAATAAGCCATGTTTGAAATCCACCCTGTTAAGA
CTGTGAATCGTTGGGGAAAGAGTATGAGATCCTGCT
1
10
20
100
110
120
130

Figure S5. Effect of PNA addition on *arnC* translation.

(A) INRI-seq identifies an RPF peak within *arnC* belonging to an in-frame alternative start codon. The binding site of *acpP*-PNA is indicated.

(B) INRI-seq RPF distribution around the annotated *arnC* TIS.

(C) INRI-seq RPF distribution around the alternative in-frame start codon of *arnC*.

Gray and bold, start codon. Yellow and bold, alternative start codon. Orange, SD sequence.

Figure S6

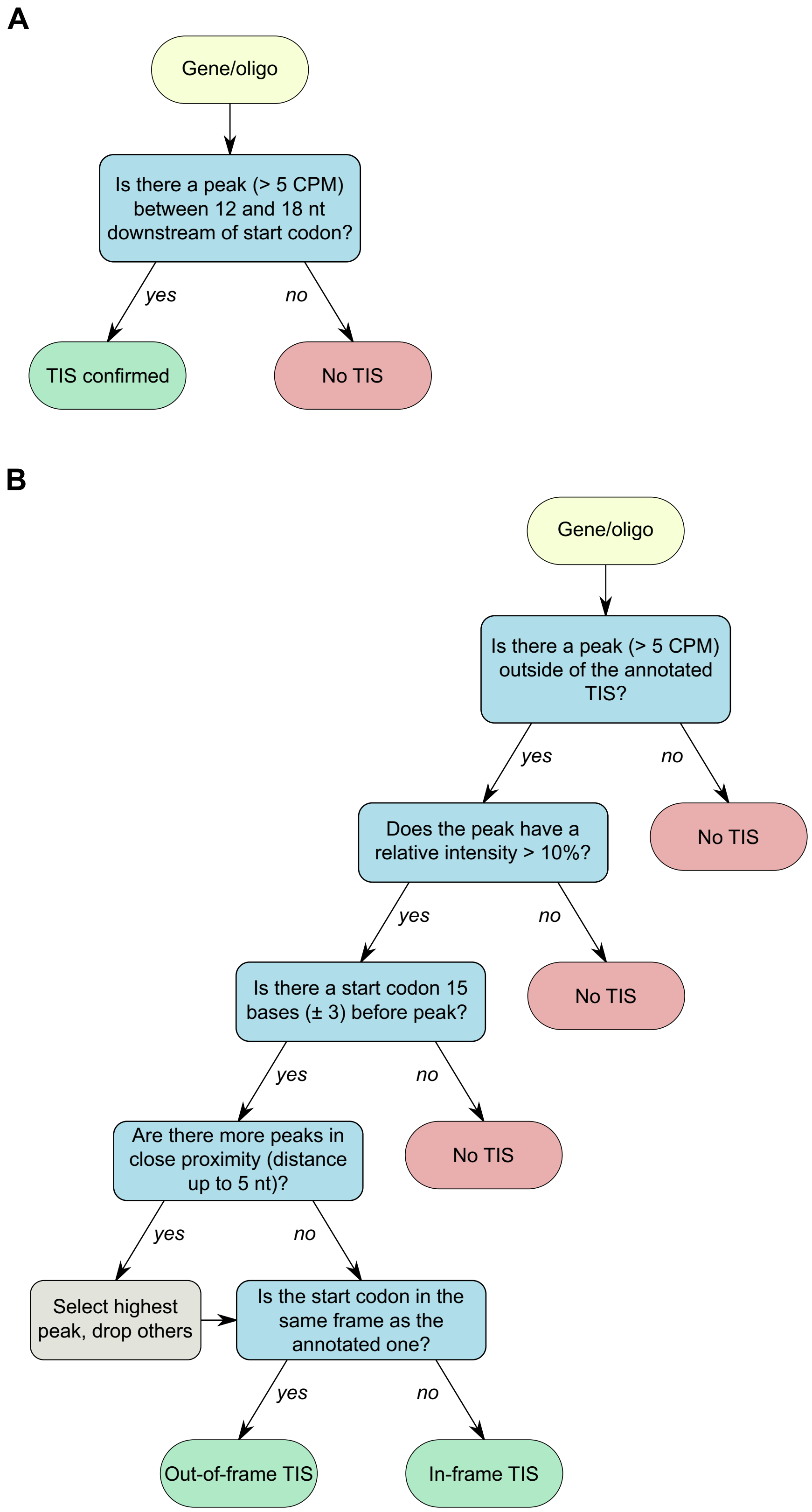
A
Gene/oligo
Is there a peak (> 5 CPM) between 12 and 18 nt downstream of start codon?
yes
no
TIS confirmed
No TIS
B
Gene/oligo
Is there a peak (> 5 CPM) outside of the annotated TIS?
yes
no
No TIS
Does the peak have a relative intensity > 10%?
yes
no
No TIS
Is there a start codon 15 bases (± 3) before peak?
yes
no
No TIS
Are there more peaks in close proximity (distance up to 5 nt)?
yes
no
Select highest peak, drop others
Is the start codon in the same frame as the annotated one?
yes
no
Out-of-frame TIS
In-frame TIS

Figure S6. Overview of TIS analysis.

(A) Analysis of annotated TISs.

(B) Analysis of putative new TISs.
