# Supplementary material for: INRI-seq enables global cell-free analysis of translation initiation and off-target effects of antisense inhibitors
Source: Nucleic Acids Res. 2022 Oct 14;50(22):e128. doi: 10.1093/nar/gkac838 (PMC9825163; doi:10.1093/nar/gkac838)
Supplement: gkac838_Supplemental_Files [file gkac838_supplemental_files.zip › Supplementary Table Legends.pdf]

SUPPLEMENTARY TABLE LEGENDS

INRI-seq enables global cell-free analysis of translation initiation and off-target effects of antisense inhibitors

Jens Hör, Jakob Jung, Svetlana Đurica-Mitić, Lars Barquist & Jörg Vogel

Table S1. rRNA sequences targeted for depletion.

A total of 24 rRNA regions were found to be enriched in INRI-seq without rRNA depletion (Supplementary Figure S1C, D). The sequences belonging to these regions are shown in this table and were targeted for depletion using a CRISPR approach (see Methods).

Table S2. INRI-seq data for annotated TISs.

This table shows the RPF peak data of the five INRI-seq replicates (Repl.) for each annotated TIS of *E. coli* K-12 MG1655 (NC_000913.3). For an annotated TIS to be considered identified, 2 of the 5 replicates were required to have RPF values > 5 (see Methods). For comparison, *in vivo* Ribo-RET data are shown (Meydan et al., Mol Cell, 2019). Start position represents the position of the first nucleotide of the gene within the *E. coli* genome. Strand represents whether the gene is encoded on the leading (1) or lagging strand (-1).

Table S3. INRI-seq data for non-annotated TISs.

This table shows the highest RPF peak among the five INRI-seq replicates for each putative new TIS and in how many of the five replicates the TIS was detected. For a putative new TIS to be considered identified, 2 of the 5 replicates were required to have RPF values > 5 (see Methods). For comparison, *in vivo* Ribo-RET data are shown (Meydan et al., Mol Cell, 2019). Start position represents the position of the first nucleotide of the putative new TIS within the *E. coli* genome. Start codon represents the type of start codon of the putative new TIS. nt from annotated start codon represents the distance of the first nucleotide of the putative new TIS from the first nucleotide of the annotated TIS. Frame represents whether the putative new TIS lies in frame or out of frame to the gene it was identified in; out-of-frame TISs without a stop codon within the ORF of the gene were labeled as undefined. Strand represents whether the gene is encoded on the leading (1) or lagging strand (-1).

Table S4. Transcript abundance within the synthetic transcriptome.

This tables shows the transcript abundance for each gene within the synthetic transcriptome in counts per million (CPM).

Table S5. Targets of *acpP*-PNA and *acpP*-PNA-scr.

The first sheet of this table shows all targets of *acpP*-PNA with up to one mismatch. The second sheet shows all target genes, whose ribosome binding sites (RBS) can be bound

by *acpP*-PNA with up to one mismatch. The third sheet shows all targets of *acpP*-PNA with up to one mismatch.

Table S6. Oligo pool used for the synthetic transcriptome.

This table shows the single-stranded DNA oligonucleotides that were used to create a synthetic transcript for each annotated, protein-coding gene of *E. coli* K-12 MG1655 (NC_000913.3) (see Methods).

Table S7. Oligonucleotides used in this study.

Sheet one shows the DNA oligonucleotides used in this study as well as their purposes. The second sheet shows the sequences of the used PNAs.
